# Functional Plasticity after Unilateral Vestibular Midbrain Infarction in Human Positron Emission Tomography

**DOI:** 10.1371/journal.pone.0165935

**Published:** 2016-11-08

**Authors:** Sandra Becker-Bense, Hans-Georg Buchholz, Bernhard Baier, Mathias Schreckenberger, Peter Bartenstein, Andreas Zwergal, Thomas Brandt, Marianne Dieterich

**Affiliations:** 1 Department of Neurology, University of Munich, Munich, Germany; 2 German Center for Vertigo and Balance Disorders-IFB, University of Munich, Munich, Germany; 3 Department of Nuclear Medicine, Johannes Gutenberg-University, Mainz, Germany; 4 Department of Neurology, Johannes Gutenberg-University, Mainz, Germany; 5 Department of Nuclear Medicine, University of Munich, Munich, Germany; 6 Munich Cluster of Systems Neurology (SyNergy), University of Munich, Munich, Germany; 7 Institute for Clinical Neuroscience, University of Munich, Munich, Germany; UMR8194, FRANCE

## Abstract

The aim of the study was to uncover mechanisms of central compensation of vestibular function at brainstem, cerebellar, and cortical levels in patients with acute unilateral midbrain infarctions presenting with an acute vestibular tone imbalance. Eight out of 17 patients with unilateral midbrain infarctions were selected on the basis of signs of a vestibular tone imbalance, e.g., graviceptive (tilts of perceived verticality) and oculomotor dysfunction (skew deviation, ocular torsion) in F18-fluordeoxyglucose (FDG)-PET at two time points: A) in the acute stage, and B) after recovery 6 months later. Lesion-behavior mapping analyses with MRI verified the exact structural lesion sites. Group subtraction analyses and comparisons with healthy controls were performed with Statistic Parametric Mapping for the PET data. A comparison of PET A of acute-stage patients with that of healthy controls showed increases in glucose metabolism in the cerebellum, motion-sensitive visual cortex areas, and inferior temporal lobe, but none in vestibular cortex areas. At the supratentorial level bilateral signal decreases dominated in the thalamus, frontal eye fields, and anterior cingulum. These decreases persisted after clinical recovery in contrast to the increases. The transient activations can be attributed to ocular motor and postural recovery (cerebellum) and sensory substitution of vestibular function for motion perception (visual cortex). The persisting deactivation in the thalamic nuclei and frontal eye fields allows alternative functional interpretations of the thalamic nuclei: either a disconnection of ascending sensory input occurs or there is a functional mismatch between expected and actual vestibular activity. Our data support the view that both thalami operate separately for each hemisphere but receive vestibular input from ipsilateral and contralateral midbrain integration centers. Normally they have gatekeeper functions for multisensory input to the cortex and automatic motor output to subserve balance and locomotion, as well as sensorimotor integration.

## Introduction

The vestibular system is organized bilaterally with ascending and descending pathways; they cross three times in the brainstem and at least once in the hemispheres between vestibular cortex areas [1,2]. The bilateral structure is the key to its sensory, sensorimotor, and higher vestibular cognitive functions, and–most importantly for the neurologist–to its various disorders [3]. Relevant structures in the brainstem circuitry for processing vestibular information are the vestibular nuclei (VN) in the pontomedullary brainstem and an assembly of nuclei for eye-head integration in the midbrain tegmentum including the interstitial nucleus of Cajal (INC), the rostral interstitial nucleus of the medial longitudinal fasciculus (riMLF), the oculomotor nucleus with paired and unpaired subnuclei, and the posterior commissure (PC). The INC projects to the neck and the rest of the spinal cord; it is presumably involved in motor control in the sagittal plane and especially in the control of vertical and oblique eye movements. Strokes at pontomedullary and midbrain levels frequently elicit vestibular disorders that typically recover within weeks to months; they have a time course comparable to that of acute peripheral vestibulopathy. Unilateral medullary infarctions of the VN (Wallenberg’s syndrome) and paramedian strokes of midbrain basilar artery branches cause typical vestibular and ocular motor syndromes. The vestibular dysfunction in Wallenberg’s syndrome is characterized by lateropulsion of eyes and body, skew deviation, eye torsion, as well as tilts of the head and perceived verticality, i.e., the ocular tilt reaction (OTR) [4,5]. Unilateral midbrain lesions of the INC also typically manifest with OTR [6,7]. Thus, the two clinical vestibular syndromes, although elicited at different levels of the brainstem, share certain signs and symptoms. They differ, however, in the direction of tilts of the OTR, which is ipsiversive at the pontomedullary and typically contraversive at the mesencephalic level because of the pontine crossing of the graviceptive pathways [7]. A major structural difference between the VNs and the midbrain nuclei is that right and left VNs are anatomically clearly separated, whereas the paramedian midbrain nuclei seem to form a structural and presumably also functionally connected neuronal assembly [8]. These structures have tight reciprocal as well as commissural connections to their contralateral nuclei; some of them are excitatory, others inhibitory [9], ensuring in part the close vertical coupling of the two eyes and their stabilization.

The clinical course of both vestibular brainstem disorders, either at the pontomedullary or midbrain level, is similar. The tone imbalance recovers within weeks as a result of the so-called central vestibular compensation. Central compensation of vestibular tone imbalance elicited by acute unilateral peripheral vestibular failures has been extensively studied in animals [10–12] and humans [13–21]. So far central compensation of a unilateral *central* lesion of the VN has been documented in humans in a fluordeoxyglucose (FDG)-PET study on Wallenberg’s syndrome [22]. In the acute phase the contralateral VN and the cerebellum bilaterally were shown to be activated, while the visual cortex was down-regulated. After functional recovery 6 months later these signal changes had normalized in the follow-up PET, suggesting that the compensatory mechanism was mainly mediated by brainstem and cerebellar structures.

The aim of the present study was to determine whether unilateral ischemic lesions of vestibular midbrain structures elicit different compensatory mechanisms than medullary VN lesions. Since the VN and INC subserve different subfunctions of vestibulo-spinal and vestibulo-ocular reflexes (VN) and eye-head coordination for orientation in space (INC), the consequences for thalamic and cortical perceptual functions may differ.

On the basis of this anatomical and clinical background, the following questions were posed about central compensation or substitution after acute unilateral midbrain infarctions:

- Which vestibular areas are involved–areas of the cortical network and/or brainstem-cerebellar loop areas?
- Are the other sensory systems–somatosensory, visual, auditory–involved in central compensation or substitution processes?
- Which possible mechanisms—neuronal up- or downregulation–take place in the areas involved?

By answering these questions, we aim to disclose potential compensatory strategies, which seem to differ depending on the vestibular lesion site. We, therefore, investigated the resting state of patients with acute unilateral vestibular midbrain stroke twice by FDG-PET: once in the acute phase, and a second time 6 months later after clinical recovery. We performed group subtraction analyses and in addition compared the patients’ imaging data with those of age- and gender-matched healthy controls. Voxelwise lesion-behavior mapping (LBM) was performed on the structural MRI data of the patients in order to determine the exact extent of the acute lesion site and to check whether vestibular structures are affected on analogy to the data of larger sample sizes [23,24].

## Material and Methods

### Patients

Seventeen patients with acute unilateral midbrain (meso-diencephalic) infarctions (7 right-sided, 10 left-sided) were consecutively recruited from the stroke unit (mean age 65.6 years; 12 males). The diagnosis was based on key symptoms and morphological signs of a stroke in the MRI scans, a neurological and neuro-otological examination including neuro-orthoptic assessment. All patients underwent a standardized neuro-otological workup including recordings of the head-impulse test, caloric warm and cold water irrigation, and examination of the saccadic, smooth pursuit, and optokinetic ocular motor systems as well as fixation suppression of the vestibulo-ocular reflex (e.g., binocular electro- or video-oculography). In accordance with international standards vestibular paresis was defined as a reduced peak slow phase velocity of the caloric nystagmus ≤ 5°/s or > 25% asymmetry between the right-sided and the left-sided vestibular caloric responses; this was calculated with the formula of Jongkees [25].

To determine the signs of otolith dysfunction and vestibular tone imbalance in the roll plane, i.e., the components of an ocular tilt reaction (OTR), such as head tilt, skew deviation, ocular torsion, and perceptual tilts [6], examinations with Frenzel’s glasses, fundus photography by a laser ophthalmoscope, and standardized testing for tilt of the perceived subjective visual vertical (SVV) were performed. The adjustment of the SVV was measured monocularly and binocularly in an upright body position while the patient looked into a hemispheric dome (60 cm in diameter) covered with a random pattern of colored dots, containing no clues about gravitational orientation. The patient had to adjust a central test target (straight line in the center of the dome) from a random offset position to the perceived vertical using a potentiometer. SVV is determined by calculating the means of 10 adjustments (pathological deviation >2.5°) [7]. A complete ocular tilt reaction (OTR) is the combination of all three signs of vestibular tone imbalance in the roll plane [26].

None of these patients had a history of previous central nervous system disorders, cochlear or vestibular disorders; none took vestibular sedatives.

Thin-slice MRI (3 mm; voxel size 0.5 x 0.5 x 3 mm^3^) T2-weighted fluid inversion-recovery sequences within 2 days after symptom onset were used to verify the acute brainstem lesions by diffusion-weighted images (1.5T clinical MRI scanner, Siemens, Germany). The MRI images were independently analyzed by two neurologists. Only unilateral infarctions that did not cross the midline were included.

In the PET study only the subgroup of eight patients with vestibular signs, pathological SVV tilts, which indicate a graviceptive vestibular tone imbalance in the roll plane, was included (6 males; mean age 64.1 years; 4 right- and 4 left-sided infarctions).

Lesion behavior mapping of the MRI data was additionally used to verify the extent and site of the lesions, e.g., the exact midline zoning of the infarctions and demarcation for the two patient groups. Furthermore, it was used to check if the lesion site in our patients fits that of larger patient samples reported in the literature [23,24]. This procedure ensured a homogeneous sample of patients with typical midbrain lesions that cause unilateral vestibular dysfunction.

Patients underwent resting-state FDG-PET and neurological as well as neuro-otological examinations at two time points: first during the acute stage and second 6 months later after clinical recovery (n = 5).

### Ethics statement

The study involving human participants was conducted in accordance with the ethical standards of the institutional and national research committee and with the 1964 Helsinki declaration and its later amendments or comparable ethical standards. The study was approved by the local Ethics Committee and Radiation Protection Authorities (FDG-PET: IRB number 837.131.03(2793), 7th May 2003; MRI and neurophysiology: IRB number 837.406.04(4564), 15th December 2004). Informed written consent was obtained from all individual participants included in the study. Participant written consent was documented on a standard form approved by the local Ethics Committee.

### PET measures, data acquisition, and image reconstruction

The patients with vestibular lesion signs, i.e., pathological SVV tilts, underwent two resting state PET scans following injection of 150 ± 20MBq FDG in a standardized manner [27] in an ECAT Exact PET Scanner (Siemens/CTI, Knoxville) while they lay supine in a quiet, darkened room with eyes closed and without any artificial stimulation: (A) during the acute stage of brainstem infarction (mean on day 4.4 after symptom onset, range: day 2 to 7) and (B) 6 months later after clinical recovery, corresponding to that in earlier studies on peripheral vestibular (mean day 6.6, respectively 6.8) [17,19] and medullary lesions (mean day 8) [22]. The interval between symptom onset and first PET scan varied between the patients for logistical reasons (e.g., availability of the tracer or scanning time, transport logistics). The time frame of 6 months was chosen for the second PET scan, since the clinical vestibular deficit had recovered by that time, and this time frame closely corresponds to that used in earlier studies on peripheral and medullary vestibular lesions [14,19,22].

The two examinations were performed at the same time of the day to minimize influences of circadian variability on glucose metabolism [28]. On the examination day patients were asked to fast for at least 8 hours before the PET study, but were allowed free access to unsweetened drinks. To obtain transaxial images approximately parallel to the intercommissural line (AC-PC line), the patient was positioned on the scanner`s bed with the canthomeatal line parallel to the detector rings. The emission scan started 30 minutes after 18-FDG injection and continued for 20 minutes in a three-dimensional acquisition mode. The patients remained in one bed position, since the scanner has an axial field of view of 16.2 cm, covering the whole brain. Attenuation correction was calculated using a computerized threshold limit routine to define an isodensity contour of the maximum cerebral activity per pixel. The exact position of an iso-intensity density contour was checked visually slice-by-slice and eventually corrected manually. Forty-seven transversal slices, each 3.375-mm-thick, were reconstructed using filtered back-projection with a ramp and a 4-mm Hanning filter. The images had a transaxial resolution of 6.0 mm in the center of the field-of-view with full width at half maximum (FWHM) [29].

### PET statistical analysis

Data analysis was performed with Statistical Parametric Mapping software (Statistical Parametric Mapping, Wellcome Department of Cognitive Neurology, London, UK, http://www.fil.ion.ucl.ac.uk/spm). The images were realigned, stereotactically normalized into the standard anatomical space by means of linear and nonlinear transformation [30], and smoothed with a three-dimensional Gaussian filter using a 12-mm full-width at half-maximum kernel. To have all infarctions on the same side, the data of patients with right-sided brainstem infarction were flipped before normalization to the left. The effect of the two different stages of vestibular midbrain infarction on regional cerebral 18-FDG activity was estimated according to the general linear model [31]. After proportional scaling of all scans to mean global cerebral activity [32], t statistical parametric maps were calculated for categorical comparisons on a voxel-by-voxel basis using a pooled variance estimated from the whole-brain gray matter [33]; the values were expressed as Z-scores.

Furthermore, patient PET data were compared with those of 18 healthy controls (mean age 59.4 years) who had been scanned earlier under identical resting state conditions (standardized procedure) using a two-sample t-test. This additional analysis was calculated, since no PET scan prior to the infarction was available for the patients. It allowed us to check the activation patterns induced by the vestibular tone imbalance in the acute stage per se (serving as substitution baseline). For correlation analyses with glucose metabolism the individual SVV parameters in the acute stage (in °) were entered as covariates into the design matrix (n = 8). Age and gender were added to the regression analysis. A statistical threshold of p<0.001 (uncorrected) and a minimal cluster size > 10 voxels were always applied. These methods of standardized PET data acquisition, image reconstruction, and statistical analyses was chosen for optimal comparability of the data to those of earlier FDG-PET studies on vestibular syndromes [14,19,22,27].

For anatomical localization of clusters, the MNI coordinates were transformed into the Talairach space using the mni2tal tool provided by CBU Imaging wiki (http://imaging.mrc-cbu.cam.ak.uk). For most correct labelling of brainstem and cerebellar clusters the extended version of the cytoarchitectonic brainstem atlas of Olszewski and Baxter [8], the MRI atlas of the human cerebellum [34], and the stereotaxic atlas of Schaltenbrand and Wahren [35] were additionally used. However, the single components of infratentorial clusters were elaborated with extreme caution because of the limited spatial resolution of PET. Due to partial flipping of the patient data, the terms “ipsilateral” or “contralateral” to the lesion side rather than “right” or “left” are used throughout the manuscript.

### MRI lesion-behavioral mapping (LBM) to control the extent and site of the midbrain lesions

Lesion behavior mapping associates the location of brain injury with the resulting symptoms. Thus, LBM associates statistically the location of brain injury with the resulting symptoms such as the pathological tilt of SVV.

The MRI dataset was first prepared using the isolation algorithm of the SUIT toolbox [36,37]. Afterwards the normalization algorithms provided by SPM 5 and the SUIT toolbox implemented in SPM5 were applied. For the normalization procedure, the isolation map was used as a mask based on the SPM interface. The lesions were delineated directly on the individual, normalized MRI scans with MRIcron software [38]. Finally, the extent of the lesion shapes was compared with the original MRI dataset by a second neurologist.

A subtraction analysis (n = 17 patients) of the anatomical MRI images was calculated to test whether the lesion site of patients with significant tilts of the SVV (n = 8) differ from that of patients without SVV tilts (n = 9) and to verify the anatomical midbrain structures involved in vestibular processing compared to earlier data in larger sample sizes [23].

Quantitative subtraction analysis reflects the relative frequency of damage. For subtraction analysis all images of patients with right-sided lesions were flipped to the left. To identify the various structures affected, the lesions were compared with those in the extended version of the cytoarchitectonic brainstem atlas of Olszewski and Baxter [8] as well as with those in the MRI atlas of the human cerebellum [34], and stereotaxic atlas of Schaltenbrand and Wahren [35]. Since the INC and the riMLF cannot be identified in MRI, neighboring structures were used, such as the oculomotor and trochlear nucleus or red nucleus [39]. In addition, due to the lack of probabilistic maps of the brainstem, the INC and riMLF were identified with the help of the data reported by Büttner-Ennever *et al*. [40] and Horn *et al*. [9]. Slices correspond to coordinates in MNI space.

## Results

### Patient data

Acute stage: all eight patients with pathological SVV tilts exhibited a typical vestibular midbrain syndrome with unstable stance and gait (n = 8) associated with a tendency to fall to the side (6 contralesional, 2 ipsilesional), as well as transient double vision (n = 6). Only one patient reported a transient rotatory vertigo for a few minutes and two patients, a prolonged discrete to-and-fro vertigo, whereas five patients did not report vertigo/dizziness at all (no reduced consciousness). Central ocular motor deficits in the vertical direction were present in all patients (6 vertical gaze palsy, 7 impaired vertical saccades, 6 impaired vertical optokinetic nystagmus). Spontaneous nystagmus was seen transiently in only one acute-stage patient (horizontal); it was not detected in any of the patients at the time of the first PET scan. Tonic effects of vestibular dysfunction in the roll plane in terms of an in-/complete ocular tilt reaction were found in five patients (3 head tilt, 3 skew deviation, 3 ocular torsion; 2 complete and 3 incomplete OTR). All eight patients showed significant binocular SVV tilts (mean 11.0°; SD 3.6°; 2 ipsiversive, 6 contraversive). Three patients exhibited an ipsilateral oculomotor nerve palsy. Furthermore, patients had dysarthria (n = 6), contralateral hemiparesis/-ataxia (n = 6), or upbeat nystagmus (n = 1). Recordings of the caloric testing and head-impulse test were normal in all patients; this was to be expected in central lesions at midbrain level. The detailed results of the clinical and psychophysical examinations of the patients in the acute stage are listed in Table 1.

**Table 1:** Patient data in the acute stage of midbrain infarction.

patients	lesion side	symptoms			ocular motor signs					ocular tilt reaction				SVV	fall / body tilt	clinical findings
		unstableness of stance/gait	vertigo	double vision	gaze palsy	impaired saccades	impaired OKN	SPN GEN	impaired smooth pursuit	head tilt	skew deviation	ocular torsion	OTR			
1 D.J.	R	+	initially rotatory	+	-	-	-	- SPN L/R	-	L	R>L	-	incomplete	-9.3°	left	hemiparesis L, dysarthria, ptosis R
2 D.KH.	L	+	to-and-fro	+	-	left + down	-	- SPN L/R	+	-	-	n.d.	-	11.3°	right	INO L, hemiparesis R, hemiataxia R, ptosis L, lesion CN III L
3 G.K.	R	+	-	+	vertical	vertical	vertical	- SPN -	+	R	L>R	R	complete	29.0°	right	
4 M.G.	R	+	-	-	vertical	vertical	vertical	- SPN L	+	-	-	n.d.	-	-18.6°	left	hemiparesis L, hemiataxia L, lesion CN III R, ptosis R, dysarthrophonia, dysphagia
5 M.H.	L	+	-	+	vertical	vertical	vertical	- SPN all	+	R	L>R	R	complete	8.0°	right	Hemihypesthesia, face L, upbeat-nystagmus, dysarthria
6 S.U.	L	+	-	-	vertical	all	vertical > horizontal	- SPN all	+	-	-	R	incomplete	7.5°	left	hemiparesis R, hemiataxia R, lesion CN III L, ptosis L, dysarthria
7 T.H.	R	+	-	+	vertical	vertical	vertical	- SPN vertical	+	-	-	-	incomplete	7.5°	left	hemiparesis/-ataxia L, dysarthria, SPN left
8 V.E.	R	+	to-and-fro	+	vertical	vertical	vertical	- SPN L/R	+	**-**	-	-	-	-4.2°	right	dysarthrophonia
Σ	5 R / 3 L	8	1 rotatory, 2 to-and-fro	6	6	7	5	0 SPN, 7 GEN	7	3	3	3	5	contra-/ ipsiversive 6/2	contra-/ ipsilesional 5/3	

Key symptoms and objective clinical signs (identified by neurological and neuro-otological examination including neuro-orthoptic assessment) in the eight patients with pathological SVV tilt and PET due to an acute unilateral midbrain infarction.

Abbreviations: CN = cranial nerve; GEN = gaze-evoked nystagmus; L = left; INO = internuclear ophthalmoplegia; OKN = optokinetic nystagmus; OTR = ocular tilt reaction; R = right; SPN = spontaneous nystagmus; SVV = subjective visual vertical; n.d. = not done; (+) = present; (-) = not present; (>) = above.

The vertical extent of the infarction in the MRI varied: All were clearly restricted to one side, four lesions were located in the mesencephalic brainstem, four lesions reached partly up into the lower thalamus. Of the latter, two patients showed ipsiversive tilts of the SVV; here the infarction partly affected inferior thalamic nuclei such as the nuclei endymalis thalami or parafascicularis thalami. These inferior parts of the thalamus convey parts of the vestibular graviceptive pathways [24].

The careful, standardized interdisciplinary workup revealed cardiac or arterio-arterial embolism as the most probable etiology of infarction in four PET patients; in the other four patients the exact etiology remained unknown. The laterality quotient for handedness according to the 10-item inventory of the Edinburgh test was +100 in six and +80 in two patients; thus the patients were strongly right-handed [41,42].

Follow-up: Six months after symptom onset five patients agreed to a second clinical examination and FDG-PET. All five had significantly recovered; three reported a slight unsteadiness of gait and two an intermittent lightheadedness. At this stage none of them showed spontaneous nystagmus, gaze palsy, head or body tilt, ocular torsion, skew deviation, or SVV tilts anymore. Neuro-orthoptic examination revealed discrete residual central ocular motor signs in the vertical direction in only three patients (slightly slowed or hypometric saccades, impaired optokinetic nystagmus or vertical smooth pursuit). Only one patient showed a latent hemiparesis/-ataxia.

The two patient groups (with and, respectively, without a second PET) showed no significant differences in mean age, clinical and neuro-otological signs at symptom onset, or days until first PET.

### Anatomical data

**Fig 1:**
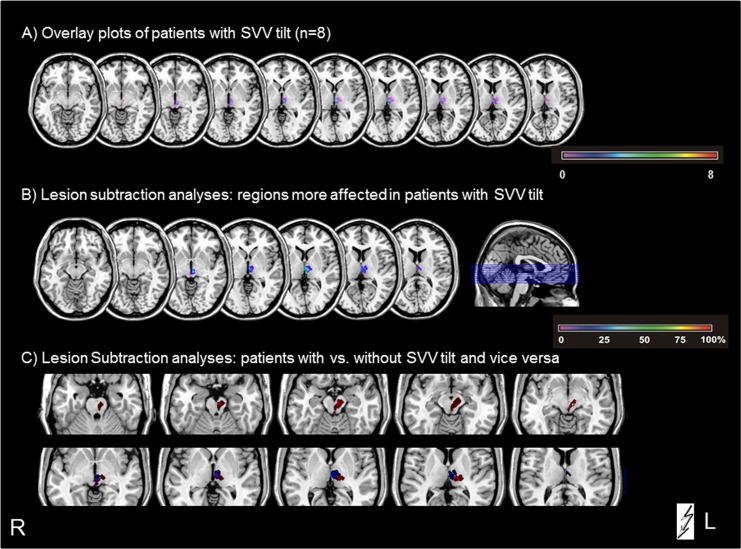
Overlay plots and subtraction analyses of the MRI LBM in midbrain lesions. A) Overlapping lesion plots of the patients with pathological SVV tilt show voxels primarily in the rostral paramedian mesencephalic-diencephalic regions and illustrate the exact midline zoning of the lesions. B) Subtraction analyses of patients with SVV tilt versus patients without tilt. The percentage of overlapping lesions in the patients with tilt after subtraction of the controls is illustrated by different colors coding for increasing frequencies—from violet (1%) and increasing to dark red (100%). C) Overlay plots of the subtraction analyses of patients with vs. without SVV tilt (indicated in blue) and vice versa (indicated in red), illustrating that there is no overlap of the lesion sites at the mesencephalic brainstem level.

The LBM overlay plots (Fig 1) confirmed the exact midline zoning of the infarctions, essential for functional interpretation of the PET pattern. The LBM subtraction analyses between the patients with versus those without SVV tilts showed that in patients with SVV tilts the voxels preferentially affected rostral paramedian mesencephalic-diencephalic regions, whereas the LBM analysis of patients without SVV tilts and the opposite subtraction contrast of patients without versus those with SVV tilts revealed voxels in more laterally located mesencephalic regions and regions ipsilesionally downward toward the pontine brainstem (Fig 1). The patient groups with and without tilt of SVV do not show any substantial lesion overlap. Consequently, it was possible to conclude that in patients with SVV tilts the voxels affecting the region of the INC and the riMLF were damaged by up to 70% more frequently; this corresponds to findings in earlier LBM studies [23]. Furthermore, plots of the subtraction analyses showed no overlap of the lesion sites.

### FDG-PET data

**Fig 2:**
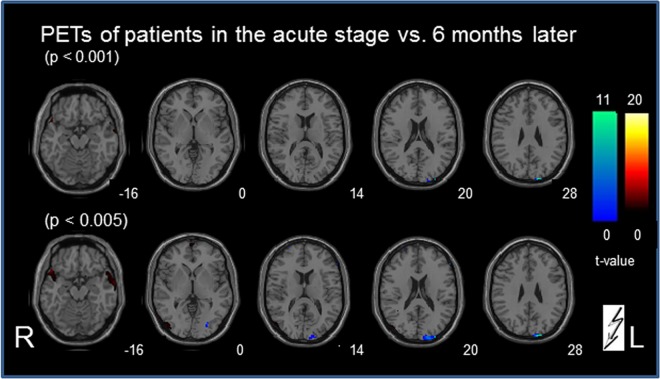
Statistical group subtraction analysis (paired t-test) of the PET scans in patients with vestibular midbrain lesions. The contrast of the PET scan at the acute stage vs. a second scan 6 months later after recovery (indicated in red) revealed at a threshold of p < 0.001 only small bilateral inferior temporal signal differences that partly merged into the inferior-most insula region bilaterally at a lowered significance level of p < 0.005. The inverse contrast recovery vs. acute stage (indicated in blue) showed at p < 0.001 only one signal cluster in the ipsilesional primary visual cortex that expanded at a lowered significance level (p<0.005). At this lowered significance level one additional ipsilesional lateral visual cluster became evident. No signal changes were seen in the cerebellum or other cortical areas for both contrasts, even at the lowered threshold. The numbers on the bottom right of each slice indicate the z-level of the Talairach space.

Paired t-test (Fig 2): The contrast between the PET A and PET B showed small bilateral, inferior temporal signal changes (parts of the inferior/medial temporal gyrus, Brodmann area (BA) 20/21; the superior temporal gyrus, BA 38; and the limbic lobe parahippocampal gyrus / uncus (BA 20) (at a threshold of p < 0.001). At a lowered significance level of p < 0.005 these inferior temporal clusters partly merged into the most inferior insula region (middle temporal gyrus) bilaterally. At this significance level additional small clusters were seen contralesionally in the lateral middle occipital/ inferior/medial temporal cortex (partly motion-sensitive area MT/V5; BA 19/37; 62 voxels), and in the superior frontal gyrus (BA 6, x/y/z = 0/66/2, 46 voxels and 2/-6/72, 71 voxels; BA 8; 4/38/56, 25 voxels) (Fig 2). No clusters were observed in the cerebellum or in known vestibular cortical areas, even at the lowered threshold p<0.005.

Conversely, the subtraction analysis recovery vs. acute stage (Fig 2) revealed only one signal cluster in the ipsilesional visual cortex (cuneus, medial occipital gyrus, BA 18/19) (at p < 0.001). At a lowered significance level (p<0.005) this single cluster expanded (z-level +14 to +32), and only one additional cluster became evident more laterally in the ipsilesional visual cortex (inferior occipital/temporal gyrus, BA 19). Even at this lowered threshold no signal changes were seen in the cerebellum or other cortical areas.

#### Comparison of patient data in the acute stage with that of healthy controls

**Fig 3:**
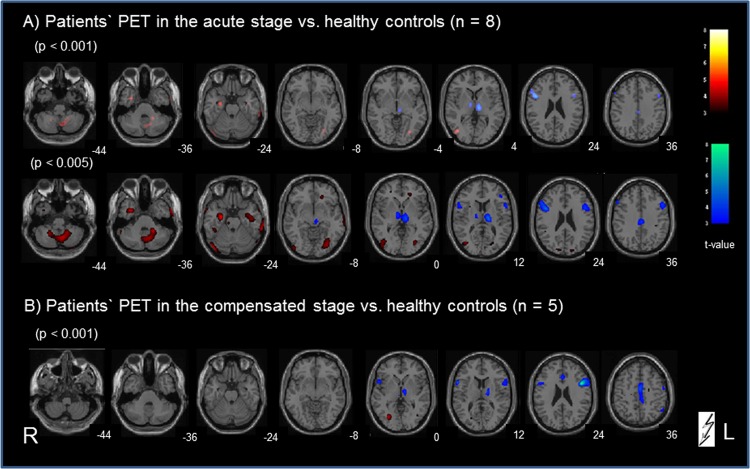
Categorical comparison of PET data in midbrain patients vs. age-matched healthy controls. A) for the acute stage vs. controls and B) after recovery vs. controls. Significant voxels in the calculation for patients > controls are indicated in red; for the inverse contrast controls > patients are indicated in blue (all p-values are uncorrected). The numbers on the bottom right of each slice indicate the z-level of the Talairach space.

PET A vs. healthy controls (Fig 3): at the cortical level significant signal differences were found bilaterally in the lateral visual cortex (middle/inferior occipital gyrus), partly merging into the motion-sensitive area MT/V5 (BA 19/37), in the inferior temporal/limbic cortex (parahippocampal/fusiform gyrus), and the central sulcus region (pre-/postcentrally; BA 4/3/6). Bilateral cerebellar clusters covered parts of the midline vermal as well as of the hemispheric structures (most likely parts of the inferior and superior semilunar, gracilis and biventer lobules, dentate nucleus, tuber, pyramid, and uvula). With lowered threshold (<0.005), all these clusters became larger in size, especially in the cerebellum, limbic, and lateral occipital areas (now including the motion-sensitive area MT/V5 on both sides; BA 19/37). Additional areas were now seen in the right medullary brainstem, the upper visual cortex bilaterally (middle occipital gyrus/cuneus; BA 18/19), and the frontal cortex bilaterally (middle frontal gyrus bordering the cingulate gyrus; right superior frontal gyrus, BA 8). All these signal clusters must be attributed to regional cerebral glucose metabolism (rCGM) increases in the acute stage.

Healthy controls vs. PET A (Fig 3): Significant signal clusters (p<0.001)—most probably due to rCGM decreases in the acute stage—were found bilaterally in known ocular motor structures (prefrontal cortex and frontal eye fields), in the inferior/middle frontal cortices (BA 9/10/44/46), in the thalamus bilaterally (ipsi- > contralesional; vestibular), and in the midline anterior cingulate gyrus (BA 24/31, vegetative). At p <0.005 these clusters expanded downward from the ipsilesional thalamic cluster to the ipsilesional midbrain, and from the cortical eye fields downward to the superior temporal gyri (BA 22). Only one additional small cluster was found in the left precentral gyrus (BA 4/6).

Since the number of patients with a second PET scan at recovery was smaller than the number of patients with an acute stage PET (n = 5 compared to n = 8) we calculated an additional subgroup analysis to make small group effects for the second PET scan analysis unlikely. The calculation of the first PET scan of only those patients who underwent a second PET scan gave a nearly identical pattern of signal increases and decreases compared to healthy controls than did that of the whole group (n = 8).

#### Comparison of patient data after recovery with those of healthy controls

PET B vs. healthy controls (Fig 3) revealed signal differences only in the middle occipital gyrus bilaterally (contra- > ipsilesional; BA 19). Even by lowering the significance level (p< 0.005) no additional positive cortical signal differences were evident. At the cerebellar level only very small signal clusters were seen in the vermis and inferior semilunar lobes. Conversely, the subtraction analysis for healthy controls vs. PET B revealed at p < 0.001 signal clusters in the ocular motor structures (prefrontal cortex and frontal eye fields), superior temporal gyrus (BA 22) and inferior/middle frontal cortices bilaterally, in the midline anterior cingulate gyrus as well as in the ipsilesional thalamus. The latter became bilateral at a level of 0.005. These patterns were similar to those in the calculation acute stage vs. controls.

#### Correlation analyses of rCGM and amount of SVV tilt

**Fig 4:**
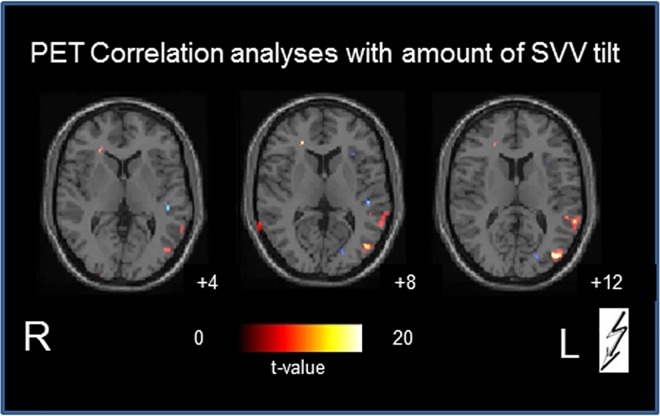
Correlation of SVV tilt and rCGM. The numbers on the bottom right of each slice indicate the z-level of the Talairach space.

In the acute phase of midbrain infarction a positive correlation of the amount of SVV tilt and the rCGM (Fig 4) was found predominantly in the ipsilesional motion-sensitive complex hMST and MT/V5 in the middle occipital/temporal cortex (BA 19/37/39), and also slightly in the contralesional inferior occipital cortex (BA 18), the middle/superior temporal gyrus (BA 21/22; ipsi- more than contralesionally), and in the contralesional inferior/medial frontal gyrus.

## Discussion

The aim of this study was to document two stages of functional plastic changes by determining the resting brain glucose metabolism in two PET scans taken after acute unilateral strokes in vestibular and ocular motor structures of the midbrain tegmentum: one in the acute phase and the other 6 months later. The first PET (mean day 4.4) not only reflected the vestibular tone imbalance at different levels of central vestibular structures but also the rapid adaptive (functional) processes to alleviate distressing symptoms such as dizziness and oscillopsia. The second PET was thought to possibly reveal long-term compensatory (structural) mechanisms that restore the balance within the bilateral central vestibular network. If this were so, it would allow inferences about structures and mechanisms involved in central compensation of a central vestibular tone imbalance. Therefore, the second scan was performed after clinical proof of perceptual and behavioral recovery.

### Lesion-induced transient activation-deactivation patterns in midbrain vs. pontomedullary infarctions

**Fig 5:**
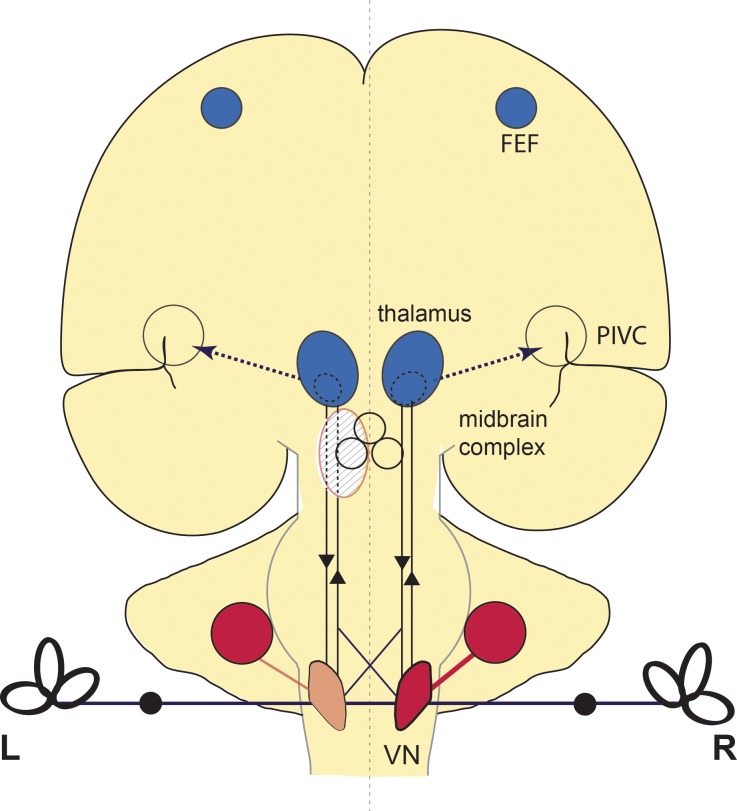
Schematic drawing of the activation pattern in acute vestibular midbrain infarction. RCGM increases (red) were seen in the contralateral (right) medullary brainstem including the VN and in the cerebellar hemispheres bilaterally. Additional increases in the cerebellar vermis cannot be found on this slice. RCGM decreases (blue) were seen in both entire thalami and areas of the frontal eye fields (FEF). The parieto-insular vestibular cortex (PIVC) showed no metabolic changes. Increases within the visual cortex are not depicted in this drawing. After recovery the infratentorial increases in the brainstem and cerebellum were largely restituted, whereas the supratentorial decreases persisted.

In the acute stage after unilateral midbrain lesions the *activated areas* included the cerebellum bilaterally (especially the vermis, and inferior semilunar lobes), parts of the temporal gyri and the secondary visual motion-sensitive areas MT/MST in the middle occipital gyri, but not the posterior insular / retroinsular region (Fig 3). This region is assumed to be a multisensory core region within the temporo-parietal vestibular network and to represent the human homologue of the parietoinsular vestibular cortex (PIVC) in monkeys [43–47]. The relevance of MT/MST activation in midbrain-lesioned patients is further underlined by the positive correlation of the amount of SVV tilt and the regional glucose metabolism (rCGM) in this area during the acute stage (Fig 5). The human MST–the homologue to the dorsal MST (MSTd) in the monkey–not only mediates optic flow but also vestibular information [48]. A comparison of vestibular spatiotemporal tuning in the macaque PIVC, the ventral intraparietal area, and the MSTd showed a gradual transformation of temporal responses, suggesting a hierarchy in cortical vestibular processing, with the PIVC being most proximal to the vestibular periphery and the MSTd being most distal. In other words, the MSTd is closest to the cortical visual input, whereas the PIVC is closest to the cortical vestibular input. Its position in the hierarchy could enable the MST to visually substitute in part for absent vestibular input. Such a possibility is further reflected by an absence of activation in the PIVC and a downregulation of the temporal areas in our study. The activations of motion-sensitive visual cortex areas may reflect a sensory substitution for the perception of motion if the cortical vestibular input is bilaterally reduced. Patients with unilateral and bilateral vestibular failure have been shown to have recourse to visual substitution for a vestibular deficit [17,49,50]. The two latter human imaging studies, for example voxel-based morphometry, demonstrated volume reductions in the vestibular cortical network (hippocampus, superior temporal gyrus) and volume increases in motion-sensitive visual cortex areas. Similar plastic cortical reorganizations take place as task-dependent morphological changes of the vestibular (anterior) and visual (posterior) parts of the hippocampal formation in professional dancers and slackliners [51].

The activations were associated with *bilateral deactivations* of the thalamic nuclei (most prominent for paramedian and posterolateral subnuclei), the frontal eye fields, the superior temporal gyri, and the anterior cingulum, e.g., all parts of the multisensory vestibular network. These PET patterns significantly differed from those obtained earlier after unilateral pontomedullary VN lesions [22]. Here only cerebellar activations were similar, but deactivations–instead of activations after midbrain lesions—were found in the visual cortex. In the latter study no deactivations were seen in the thalamic nuclei, frontal eye fields, or cingulum. A hypothetical explanation could be that the significant bilateral deactivation of the thalamic nuclei is functionally associated with deactivations of parts of the vestibular cortical network, since the paramedian and posterolateral thalamic nuclei are directly connected to the insula and have gatekeeper functions for vestibular input [52].

The functional interpretation of the course of metabolic changes elicited by pontomedullary VN lesions was that central compensation was associated with a transient hypermetabolism in ponto-cerebellar structures, which had disappeared by the stage of recovery 6 months later [22]. This transient activation of cerebellar structures, which was also observed in the current midbrain study, could be interpreted as compensatory processes that occur via brainstem-cerebellar loops. In pontomedullary VN lesions the transient hypometabolism (deactivation) of the bilateral visual cortex was attributed to a functional suppression of distressing dizziness and oscillopsia [22]. The absent deactivation of the primary visual cortex is in line with the absence of spontaneous nystagmus in acute midbrain lesions (Table 1). However, in the current study on midbrain infarctions the supratentorial deactivations have to be interpreted differently. Here the second PET scan showed bilateral deactivations of the thalamic nuclei, the frontal eye fields, superior temporal gyri and cingulum, which were largely preserved (Fig 3). The graviceptive tone imbalance (SVV and ocular motor tilts) had functionally normalized, probably due to long-term plastic reorganizations of the midbrain integration centers and the thalamic nuclei. The cingulum (cingulate sulcus visual area, CSv)—previously implicated in processing visual cues for self-motion—is activated by galvanic vestibular stimulation in human fMRI studies [53]. Thus, the deactivation of the cingulum in midbrain lesions could reflect the missing vestibular input to a site where visual and vestibular information are integrated. In parallel, deactivations of the frontal eye fields may also indicate the absent vestibular input.

### What is special about the vestibular interaction between the midbrain integration centers and the thalamic nuclei?

The ocular motor and vestibular midbrain integration centers comprise three structural and functional neuronal assemblies: (i) bilaterally separated nuclei (INC, riMLF, parts of the oculomotor nucleus), which are interconnected by interneurons crossing midline and pathways (posterior commissure); (ii) substructures that subserve bilateral eye-head coordination for up and down directions of gaze in space (riMLF); and (iii) common, unpaired subnuclei such as that for elevation of lid and eyes (parts of the oculomotor nucleus). Thus, this neuronal midbrain assembly must be considered a bilateral integration center with anatomically and functionally paired and unpaired substructures. A unilateral lesion is clinically characterized mainly by ocular motor and perceptual dysfunction, especially in the vertical roll plane [6,7,54]. Therefore, perceptual and ocular motor tilts (OTR and its components), which are indicative of a unilateral INC lesion, served as clinical inclusion criteria for the current study. OTR following lesions of the INC have been described as a “descending type” in contrast to the “ascending type” elicited by medullary VN lesions [2,4].

The vestibular and ocular motor midbrain assembly is interconnected by ipsilateral and contralateral pathways not only to the posterolateral but also to the paramedian vestibular subnuclei of the thalamus [55]. This was also demonstrated by a combined functional and structural connectivity and diffusion tensor imaging study in humans [1]. Not only posterolateral but also paramedian subnuclei of the thalamus mediate and integrate vestibular function. Thalamic astasia was initially described as caused only by dorsolateral thalamic lesions [56]. However, a case report showed that thalamic astasia and SVV tilt also occurred in a patient with paramedian/centromedian thalamic stroke [57]. This was confirmed by a recent voxel-based behavioral mapping analysis that attributed SVV tilts to lacunar strokes of both posterolateral and central paramedian thalamic subnuclei [24]. Here two distinct systems of graviceptive processing within the thalamus were described: contraversive tilts of SVV were associated with lesions of the central and posterolateral nuclei, whereas ipsiversive SVV tilts were associated with regions located more inferior, involving nuclei endymalis thalami and inferior parts of the nuclei parafascicularis thalami.

The thalami appear to be the only nuclei within the ascending and descending “rope-ladder-like” vestibular network not directly connected by a midline crossing. The absence of such a crossing hinders bilateral integration of vestibular function at the level of the right and left thalamic nuclei. This is compatible with the bilateral deactivations of the thalami secondary to a unilateral midbrain lesion of the current study. On analogy with the geniculate bodies within the visual system, the thalami can be perceived as gatekeepers of vestibular ascending projections to the various multisensory temporo-parietal cortex areas [58]. This fits the data of an H_2_^15^O-PET study of patients with unilateral thalamic infarctions who exhibited a complete lack of vestibular cortex activations within the affected hemisphere during caloric vestibular stimulation [52].

The thalami are, however, also part of the thalamo-basal ganglia-cortical loops for locomotion [59,60]. These adjust automatic patterns of posture and locomotor control to voluntary goal-directed movements while maintaining balance. The thalami provide the ascending afferent sensory information and project the motor output to the brainstem and cerebellar locomotor centers and the extended ponto-reticulo-spinal eye-head coordination network [61]. The loops operate separately at the thalamic level for both right and left hemispheres. The sensory thalamo-cortical networks also work task-dependently and may shift their sensory dominance from one modality to the other [62]. Thus, the full impact of the thalamus appears to be more than simply controlling the sensory information from the periphery and other parts of the brain to the cortex. It also plays an active role in all sensorimotor integrations [63].

Reflexive stabilization of eye and head as well as automatic locomotion at higher speed are mediated by infrathalamic brainstem and cerebellar locomotor centers [64–66]. In contrast, conscious perception of body posture and motion, goal-directed movements, and control of low-speed locomotion are mediated by suprathalamic sensorimotor mechanisms and higher cognitive vestibular functions including spatial orientation and navigation [67,68].

### How to interpret bilateral supratentorial deactivation after unilateral midbrain lesions?

A bilateral down-regulation of the thalamic nuclei persisting for more than 6 months permits alternative explanations. One could speculate that it indicates a partial disconnection of ipsilateral and contralateral vestibular sensory input to both thalami and the cortex bilaterally. Alternatively, it could reflect a discrepancy between the expected and the actually available vestibular input at the thalamic level. The latter has been proposed to explain the reciprocal inhibitory interactions between visual and vestibular cortex areas found for vestibular and visual motion stimulation [69,70]. Such a mechanism could increase the sensitivity of the thalamic nuclei for vestibular input (lowered threshold of “gatekeeping”) and/or shift the sensorial weight to the more reliable sense in case of a multisensory mismatch [69].

Is it more likely that the bilateral down-regulations represent an artificial result caused by the infarction? It has been reported that focal cerebral ischemia may elicit an inflammatory response that results in a focal increase in FDG uptake induced by activated microglia and macrophages [71]. This could result in an artificial increase in the FDG signal. A direct influence of the unilateral infarction causing a bilateral signal decrease of the thalamus is unlikely, since one would expect activation (not down-regulation) nearby the infarction. Up to now, there has been no indication in the literature of unspecific effects of small brainstem infarctions on the uptake of FDG in widespread cortical and subcortical areas. Even if this were the case, the statistical normalization to the global mean (“proportional scaling”) performed in the current SPM analyses would remove or minimize such an effect.

It cannot be totally excluded by diffusion-weighted MRI (the method of choice to evaluate irreversibly injured tissue) that the blood supply in the paramedian midbrain areas of INC/riMLF is transiently bilaterally affected in the sense of a penumbra, especially since this region often receives its vascular supply from a single unpaired artery, the posterior thalamo-subthalamic paramedian artery. However, there are good arguments that speak against a bilateral midbrain oligaemia as a relevant cause for the decrease in glucose metabolism that is widespread in several thalamic nuclei of both hemispheres: The thalamic circulation is highly developed and exists as a persistent, collateral arterial network. The vascular supply of the thalamus is ensured by four arteries (the polar, paramedian, thalamogeniculate, and posterior choroideal arteries). Thus, thalamic infarction classification is based on these territories, each of which has characteristic clinical and etiologic features (for review [72]). In view of this vascular network, it appears unlikely that an oligaemia of all territories in both thalami would occur, as seen in our study. This anatomical speciality is also thought to be the reason why bilateral thalamic infarctions are very rare.

Furthermore, a penumbra is typically associated with neurological deficits. Thus, a relevant bithalamic dysfunction would cause a disturbance of consciousness as well as severe behavioral, neuropsychological, and/or cognitive changes. This was not the case in all our patients. Thus, our patients did not show signs of significant bilateral neuronal damage within the thalamus.

In conclusion, our data support the view that the thalamic nuclei operate separately for the right and left hemispheres. They have gatekeeper functions for multisensory input and automatic motor output to subserve balance and locomotion, as well as sensorimotor integration.

One shortcoming of our study is the small number of enrolled patients. However, we preferred a strict selection of midbrain stroke patients based on MRI lesion mapping and clearly defined deficits of vestibular functions as known to be typical in midbrain lesions [2,7,73] The activation-deactivation patterns for our patient group were described at a similar significance level and in similar group sizes, making them comparable to those used in earlier FDG-PET and SPECT studies on selected patients with vestibular disorders [14,15,18,22].
